# The usual course of thorax CT findings of COVID-19 infection andwhen to perform control thorax CT scan

**DOI:** 10.3906/sag-2004-293

**Published:** 2020-06-23

**Authors:** Yasemin GÜNDÜZ, Mehmet Halil ÖZTÜRK, Yakup TOMAK

**Affiliations:** 1 Department of Radiology, Faculty of Medicine, Sakarya University, Sakarya Turkey; 2 Department of Anesthesiology and Reanimation, Faculty of Medicine, Sakarya University, Sakarya Turkey

**Keywords:** COVID-19 infection, thorax CT

## Abstract

COVID-19 infection, a highly contagious disease caused by the SARS-CoV virus, and the World Health Organization declared this increasingly spreading disease as a global public health emergency (pandemic). In the diagnosis of COVID-19, the polymerase chain reaction (RT-PCR) is considered as the reference standard test. In the early stages, thorax CT findings could be present even before the onset of symptoms, thorax CT has quite high sensitivity in COVID-19 patients with false negative RT-PCR results, and it has a great importance not only in diagnosis but also in follow up. We think that it might be beneficial for our radiologist colleagues in the early diagnosis of the imaging features of this disease, by sharing the experiences we have gained by evaluating the typical and relatively atypical CT findings regarding the natural course of the tomographic findings of COVID-19 and when to control CT.

## 1. Introduction

COVID-19 infection, a highly contagious disease caused by the SARS-CoV virus, was first reported in Wuhan, China, and the World Health Organization declared this increasingly spreading disease as a global public health emergency (pandemic) on January 30, 2020 [1,2]. The disease is transmitted by inhalation or contact with infected droplets, and the incubation period ranges from 2–14 days. Symptoms are usually fever, cough, sore throat, shortness of breath. Disease findings are mild in the most of cases who are asymptomatic; however, in some patients (usually the elderly and those with comorbidity), it may progress to pneumonia, acute respiratory distress syndrome, and multiple organ dysfunction. Case mortality rate is estimated to vary between 2%–3% [3].

In the diagnosis of COVID-19, the polymerase chain reaction (RT-PCR) is considered as the reference standard test. It has been reported that SARS-CoV-2 uses angiotensin converting enzyme-2 (ACE-2) as a cell receptor in humans and to cause pulmonary interstitial damage first and then parenchymal changes [4,5].

In Turkey, the first case was reported on 11th March, 2020 and the number of cases has increased rapidly as expected since that date, as also in our province (Sakarya). In the last 20 days until 05th April, 2020, approximately 5000 thorax computed tomography (CT) images were taken with suspicion of COVID-19 in our province and 3000 PCR tests were done and thorax CT was used as a diagnostic tool in the days when PCR test could not be performed. In this period, we think that it might be beneficial for our radiologist colleagues in the early diagnosis of the imaging features of this disease, which has become a major public health problem, by sharing the experiences we have gained by evaluating the typical and relatively atypical CT findings regarding the usual course of the tomographic findings of COVID-19 and when to control CT. 

## 2. The usual course of thorax CT findings of COVID-19 infection

Recent studies have shown that; though the diagnostic value of chest radiographs is low in the early stages, thorax CT findings could be present even before the onset of symptoms, thorax CT has quite high sensitivity (98%) in COVID-19 patients with false negative RT-PCR results, and it has a great importance in early diagnosis [6,7].

In the early period of the disease (days 1–5), the bilateral distribution of subplevral or peribroncovascular, multifocal, patchy or segmental, consolidated or unconsolidated ground-glass opacities in the peripheral and posterior sections of the lower lobes of the lungs constitute the basic CT findings of our COVID-19 cases. They were compatible with most of the previous radiological studies [4,5,7]. Ground glass opacification could be seen alone or together with inter/intralobular septal thickening (the crazy paving pattern).

Between the 5th and 10th days, thorax CT findings increased gradually in the first weeks during the usual course of the disease, and ground glass opacities in the early period started to consolidate. The ground glass opacities can be seen alone or together with inter/intralobular septal thickening, the crazy paving pattern, and rarely the halo sign has also been detected. 

Between the 10th and 15th days, the peak period of the disease, the findings in the midterm increased further and proceeded to central zones and started to show a tendency to merge and rarely the reverse halo sign and air bronchogram patterns were also observed in this period.

Between the 15th and 28th days, CT findings began to gradually decrease and these findings resolved completely without any sequelae or leaving sequelae changes such as focal ground glass opacities and reticular condensations.

### 2.1. When to perform control thorax CT scan

However, in every period; pleural effusion, pericardial effusion, lymphadenopathy, the crazy paving pattern, consolidated areas more distributed and progressed to the upper lobes, cavitation and halo findings were significant in terms of true progression and poor prognosis if the patient was clinically worsened. If these findings occur, regardless of the period of the patient, thorax CT scan should be repeated.

During all of these periods, our patients may be symptomatic or asymptomatic in the first or middle period of the disease. Therefore, thorax CT findings with massive progression may be observed in patients independently regarding the clinical findings. An important point we would like to mention here is that CT findings regress after a certain period of progression in the course of the disease. In other words, clinicians may see the progressed radiological findings if they obtain control PA chest X-ray leading to ordering control thorax CT considering falsely that there is a clinical progress. In fact, the increase of radiological findings appears as a usual progress in the course of the disease. The important point here is the patient’s clinic. If the patient is clinically well, radiologist must state that the radiological progress in PA chest X-ray is the usual course of the disease, and thorax CT should not be performed again unless there are signs of poor prognosis. Otherwise, both the patient may be exposed to unnecessary radiation, and the findings of radiological progress can be perceived as a real progression, and the patient may be given more aggressive treatments and perhaps unnecessary intubation. 

As a matter of fact, the oxygen saturations of 2 nonintubated patients in our hospital were about 85–90. There were signs of progression on the chest X-rays. However, the clinics of patients (mild to moderate respiratory distress) did not deteriorate. Therefore, the nonintubated patients state that radiological progression would not mean clinical progression. One of the patients who had a stable follow-up for about a week had a radiological and clinical improvement during this period. The patient is still hospitalized in our hospital and his clinics (and oxygen saturations are between 95%–99%) continue to improve. The other patient who was clinically well and PCR test negative while radiologically progressed was discharged with stable condition after being followed up for a while since there were no signs of clinical deterioration. The clinic of the patient who was under quarantine at home has improved with the routine controls.

In this context, we would like to remind that intubation is a risk of mortality, even in normal patients, and we would like to state that intubations performed because of the progress of radiological findings in patients with COVID-19 while the clinic is in good condition may cause loss of patients. Even if the patient’s saturation decreases down to 85%–90% and radiological findings progress while clinically well, our experience is that there is better chance for recovery in the patients who can tolerate without intubation for 1 week. 

## 3. Conclusion

The course of thorax CT findings in COVID-19 infection may be particularly different in each patient and each period. Thorax CT is a very sensitive diagnostic method that makes an important contribution to both diagnosis and determining the course of the disease in eligible patients. We suggest that if the patient’s clinic is stable and in good condition or there are no additional signs of worsening despite radiological findings, we recommend not to repeatedly scan the patient with CT as well as to avoid unnecessary medical or interventional procedures.

**Disclaimer/Conflict of interest **

This work was not supported by a specific grant.
